# Causal Temporal Diffusion Networks for Drug Repurposing in Epilepsy

**DOI:** 10.1093/bib/bbaf631.074

**Published:** 2025-12-12

**Authors:** Rishik Kondadadi

**Affiliations:** Eastview High School, Apple Valley, MN, USA

## Abstract

**Aim:**

Drug repurposing offers a promising strategy for identifying new epilepsy treatments, addressing the critical need for therapeutics in the 30% of patients who remain treatment-resistant. Current computational approaches rely on correlation-based learning from gene expression signatures but fail to capture the mechanistic complexity of drug-biological system interactions. We introduce the Causal Temporal Diffusion Network (CTDN), a deep learning architecture designed to model three fundamental biological principles: causal drug-gene relationships, temporal dynamics of cellular response, and network propagation effects.

**Methods:**

CTDN integrates three core components that capture complementary aspects of drug action: (1) a causal discovery module that identifies sparse drug-gene relationships through learned gating mechanisms with L1 sparsity regularization, reflecting the biological prior that drugs act through specific molecular pathways; (2) a diffusion module that performs 10 iterative feature transformations with learnable propagation rates, modeling how drug effects cascade through protein-protein interaction networks from direct targets to downstream pathways; and (3) a temporal dynamics module using recurrent neural networks (2-layer GRU) with multi-head attention to capture the evolution of drug response over time, learning implicit temporal patterns from the distribution of measurements across different experimental conditions. Uncertainty quantification is provided via Monte Carlo dropout (30 samples), enabling calibrated confidence estimates for predictions.

We evaluated CTDN on LINCS L1000 gene expression data comprising 53,872 drug perturbation profiles with 264 landmark genes and 128-dimensional molecular fingerprints (ECFP4). The dataset includes 3,872 profiles for 34 unique antiepileptic drugs (AEDs. Data was split using stratified sampling into training (70%), validation (15%), and test (15%) sets. We compared CTDN against six baseline methods: Random Forest, Logistic Regression, Support Vector Machines, k-Nearest Neighbors, Gradient Boosting, and LINCS Connectivity Map. All methods were evaluated on identical test sets using area under ROC curve (AUROC), area under precision-recall curve (AUPRC), precision at k (P@k), and unique AED discovery metrics.

**Results:**

CTDN achieved an AUROC of 0.895 on the large-scale LINCS dataset, substantially outperforming all baseline methods: Random Forest (0.849, 5.4% improvement), Logistic Regression (0.695, 28.8% improvement), Support Vector Machines (0.687, 30.3% improvement), and Connectivity Map (0.436, 105% improvement).

CTDN successfully identified AEDs spanning multiple mechanisms including sodium channel blockade, AMPA antagonism, synaptic vesicle modulation, and calcium channel modulation, demonstrating the model's ability to discover efficacious compounds across diverse therapeutic pathways.

Ablation studies quantified each component's contribution: removing the diffusion module decreased AUROC by 9.0% (most critical component), removing causal discovery decreased AUROC by 7.4%, and removing temporal dynamics decreased AUROC by 4.9%. All three components provide synergistic improvements, validating the importance of modeling biological causality, network propagation, and temporal dynamics.

**Conclusions:**

CTDN establishes a mechanistically-grounded approach to computational drug repurposing by explicitly modeling three biological principles: (1) sparse causal drug-gene relationships, (2) iterative network diffusion of drug effects, and (3) temporal dynamics of cellular response. The substantial improvement over traditional machine learning methods (Random Forest: +5.4% AUROC, Logistic Regression: +28.8% AUROC) and signature-based approaches (Connectivity Map: +105% AUROC) demonstrates that incorporating biological mechanism is essential for accurate therapeutic prediction. The mechanistic diversity of discovered AEDs indicates that CTDN learns generalizable principles of antiepileptic activity rather than simple chemical similarity. The mechanistic diversity of discovered AEDs shows that CTDN learns general principles of drug efficacy rather than chemical similarity. These results demonstrate that mechanistic modeling of causal relationships, network diffusion, and temporal dynamics is a practical necessity for effective drug repurposing.

**References:**

1. Kwan P, Brodie MJ. Early identification of refractory epilepsy. *N Engl J Med* 2000;342(5):314-319.

2. Pushpakom S, Iorio F, Eyers PA, et al. Drug repurposing: progress, challenges and recommendations. *Nat Rev Drug Discov* 2019;18(1):41-58.

3. Subramanian A, Narayan R, Corsello SM, et al. A next generation connectivity map: L1000 platform and the first 1,000,000 profiles. *Cell* 2017;171(6):1437-1452.

4. Lamb J, Crawford ED, Peck D, et al. The Connectivity Map: using gene-expression signatures to connect small molecules, genes, and disease. *Science* 2006;313(5795):1929-1935.

5. Cho K, van Merriënboer B, Gulcehre C, et al. Learning phrase representations using RNN encoder-decoder for statistical machine translation. *Proc EMNLP* 2014:1724-1734.

6. Vaswani A, Shazeer N, Parmar N, et al. Attention is all you need. *Adv Neural Inf Process Syst* 2017;30:5998-6008.

7. Gal Y, Ghahramani Z. Dropout as a Bayesian approximation: Representing model uncertainty in deep learning. *Proc ICML* 2016;48:1050-1059.

8. Rogers D, Hahn M.** Extended-connectivity fingerprints. *J Chem Inf Model* 2010;50(5):742-754.

9. Brueggeman L, Sturgeon ML, Martin RM, et al.** Drug repositioning in epilepsy reveals novel antiseizure candidates. *Ann Clin Transl Neurol* 2019;6(2):295-309.

